# A case of recurrent obscure gastrointestinal bleeding: Heyde’s syndrome – case report and review

**DOI:** 10.1080/20009666.2018.1470441

**Published:** 2018-06-12

**Authors:** Rajarajeshwari Ramachandran, Hakim Uqdah, Niraj Jani

**Affiliations:** a Department of Internal Medicine, Greater Baltimore Medical Center, Baltimore, MD, USA; b Department of Gastroenterology, Greater Baltimore Medical Center, Baltimore, MD, USA

**Keywords:** Heyde’s syndrome, aortic stenosis, angiodysplasias, trans-valvular aortic valve replacement, gastrointestinal bleeding

## Abstract

Gastrointestinal bleeding from angiodysplasias in patients with aortic stenosis is termed as ‘Heyde’s syndrome’. We report a case of Heyde’s syndrome successfully treated with trans-catheter aortic valve replacement.

## 1. Introduction

Heyde’s syndrome is the coexistence of aortic valve stenosis and recurrent gastrointestinal bleeding from intestinal angiodysplasias [1]. Angiodysplasias are dilated, thin walled tortuous vessels in the mucosa and submucosa of the gastrointestinal tract [2].

## 2. Case report

An 85-year-old former smoker with history of chronic obstructive lung disease, stage 1 squamous cell cancer of the right lung successfully treated with surgical resection and hypertension, was admitted to the hospital with 1-day history of melena. On physical exam, he was hemodynamically stable. He was noted to have conjunctival pallor, diminished second heart sound, delayed carotid upstroke, and an ejection systolic murmur (III/VI grade) in the aortic area, radiating to the right carotid. His hemoglobin and hematocrit were 8.2 g/dl and 25.9%, respectively. His last known hematocrit from one year ago was 34.5%. His platelets, international normalized ratio, prothrombin time, and partial thromboplastin time were within normal limits.

On week prior to the index admission, he was hospitalized with hematemesis and underwent an esophagogastroduodenoscopy (EGD) which revealed fresh blood in the duodenum, but the source of bleeding could not be identified. As he continued to have active gastrointestinal bleeding, mesenteric angiogram was done and it showed hyper-vascularity in the duodenal area, without extravasation. Subsequently, he underwent prophylactic embolization of the gastroduodenal artery and his hemoglobin stabilized.

**Figure 1.**
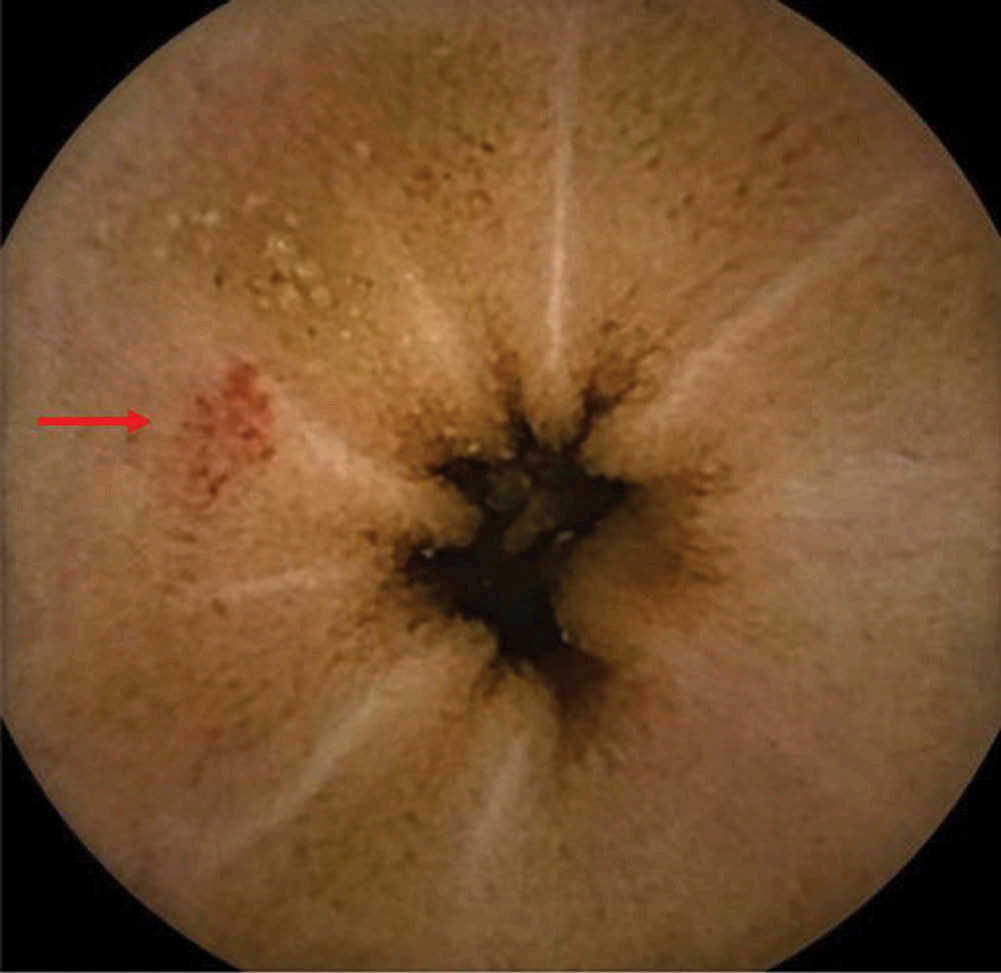
Capsule endoscopy image showing an angiodysplasia (arrow).

During the index admission, EGD and colonoscopy were performed and again the source of bleeding could not be identified. With the recurrent episodes of obscure gastrointestinal bleeds and the examination findings suggestive of aortic stenosis, Heyde’s syndrome was suspected. An echocardiogram was completed and he was found to have severe aortic stenosis with a valve area of 0.6 square centimeter and mean aortic valve gradient of 37 mmHg. Capsule endoscopy was performed and it revealed multiple arteriovenous malformations in the jejunum (Figure 1), consistent with the diagnosis of Heyde’s syndrome. His von-Willebrand factor antigens and multimeric analysis were within normal limits.

When his hemoglobin stabilized after several units of blood transfusion, he was discharged home. Due to the repeated episodes of gastrointestinal bleeding secondary to Heyde’s syndrome, aortic valve replacement was recommended to the patient. However, due to his poor functional capacity, he was deemed to be a high-risk candidate for surgical aortic valve replacement. A few months following the discharge from the hospital, he successfully underwent trans-catheter aortic valve replacement. During the 6-month follow-up after trans-catheter aortic valve replacement (TAVR), he had not required any further blood transfusions and his hemoglobin remained stable.

## 3. Discussion

Gastrointestinal bleeding from angiodysplasias in patients with aortic stenosis is referred to as ‘Heyde’s syndrome’, as this association was first described by Dr. Edward Heyde in 1958 [3]. Angiodysplasias are the most common vascular abnormalities of the gastrointestinal tract and the second leading cause of lower gastrointestinal bleeding in elderly patients [4]. They account for 1–6% of hospital admissions for gastrointestinal bleeding and are particularly common in the right colon and cecum [2]. In patients with Heyde’s syndrome, the decreased gastrointestinal perfusion secondary to severe aortic stenosis likely leads to hypoxia-induced dilation of the blood vessels and hastens the development of fixed vasodilation and genesis of angiodysplasia [5].

Von Willebrand factor (vWF) is a high-molecular-weight multimeric glycoprotein that plays an important role in primary hemostasis. The high shear stress that exists across the stenotic aortic valve in patients with Heyde’s syndrome causes a conformational change in the vWF multimers and renders them less hemostatically competent compared to their parent polymers [6]. As a result, patients with Heyde’s syndrome have acquired type 2A von Willebrand’s disease. Vincentelli et al. studied 42 patients with severe aortic stenosis and concluded that vWF abnormalities are directly related to the severity of aortic stenosis and negatively correlated with the mean transvalvular gradient across the aortic valve [7].

Routine laboratory tests performed for von Willebrand disease such as vWF antigen levels and ristocetin cofactor activity are often normal in Heyde’s syndrome [8]. In patients with normal vWF antigens such as our patient, *in vitro* closure time by platelet function analyzer (PFA-100) with adenosine diphosphate (ADP) and epinephrine can be used to confirm the diagnosis of Heyde’s syndrome [9].

Aortic valve replacement has also been shown to improve coagulation abnormalities in Heyde’s syndrome and offers the possibility of long-term resolution of symptoms [2]. Thompson et al. conducted a retrospective study of 57 patients with Heyde’s syndrome and severe aortic stenosis, treated with surgical aortic valve replacement and demonstrated that 79% of the patients had no recurrence of the gastrointestinal bleeding, during follow-up extending to 15 years [10].

Heyde’s syndrome is found in patients with co-morbidities that may put them at high risk for undergoing surgical aortic valve replacement. Trans-catheter aortic valve replacement is evolving as a suitable treatment option in these patients. In a prospective analysis performed by Sedaghat et al., it was concluded that the restoration of the high-molecular weight VWF multimer was similar in patients undergoing both surgical and trans-catheter aortic valve replacement [11].

In our review of the English medical literature, we identified 15 cases of Heyde’s syndrome treated with trans-catheter aortic valve replacement from case reports, prospective and retrospective studies [11–16]. Totally, 5/15 cases were followed up by repeating hemostaseologic testing, 7 days after the trans-catheter aortic valve replacement [11,12]. The remaining 10/15 cases had a follow-up period of 6–12 months and 9/10 cases did not experience re-bleeding.

Our case supports the use of trans-catheter aortic valve replacement in the treatment of high-risk surgical candidates with Heyde’s syndrome and highlights the importance of considering Heyde’s syndrome as a differential diagnoses in patients with recurrent obscure gastrointestinal bleeding. Further studies are needed in this area to determine the long-term effects of trans-catheter aortic valve replacement in patients with Heyde’s syndrome.
